# Resonance Rayleigh Scattering and SERS Spectral Detection of Trace Hg(II) Based on the Gold Nanocatalysis

**DOI:** 10.3390/nano7050114

**Published:** 2017-05-17

**Authors:** Huixiang Ouyang, Chongning Li, Qingye Liu, Guiqing Wen, Aihui Liang, Zhiliang Jiang

**Affiliations:** 1 Key Laboratory of Ecology of Rare and Endangered Species and Environmental Protection (Guangxi Normal University), Ministry of Education, Guangxi Key Laboratory of Environmental Pollution Control Theory and Technology, Guilin 541004, China; huixiang73@163.com (H.O.); lcn7882342@163.com (C.L.); qyliu@mailbox.gxnu.edu.cn (Q.L.); gqwen@mailbox.gxnu.edu.cn (G.W.); 2 Guangxi Colleges and Universities Key Laboratory of Regional Ecological Environment Analysis and Pollution Control of West Guangxi, College of Chemistry and Environment Engineering, Baise University, Baise 533000, China

**Keywords:** mercury ion, gold nanoparticle, nanocatalysis, resonance Rayleigh scattering, SERS

## Abstract

Mercury (Hg) is a heavy metal pollutant, there is an urgent need to develop simple and sensitive methods for Hg(II) in water. In this article, a simple and sensitive resonance Rayleigh scattering (RRS) method was developed for determination of 0.008–1.33 µmol/L Hg, with a detection limit of 0.003 μmol/L, based on the Hg(II) regulation of gold nanoenzyme catalysis on the HAuCl_4_-H_2_O_2_ to form gold nanoparticles (AuNPs) with an RRS peak at 370 nm. Upon addition of molecular probes of Victoria blue B (VBB), the surface-enhanced Raman scattering (SERS) peak linearly decreased at 1612 cm^−1^ with the Hg(II) concentration increasing in the range of 0.013–0.5 μmol/L. With its good selectivity and good accuracy, the RRS method is expected to be a promising candidate for determining mercury ions in water samples.

## 1. Introduction

Nanomaterials not only have unique optical, electrical, and magnetic properties, but also have bio-enzyme activity [1,2]. They are known as mimic nanoenzymes, and also called di-functional molecules or multifunctional molecules. Since Fe_3_O_4_ nanoparticles have been found to have intrinsic peroxidase-like activity [3], nanoenzyme have become interesting to people. They have vast application prospects with advantages of easy production process, good stability and recycling, low-cost of storage and transport, and high adaptability of heat, acid, and alkali [1,2,3,4,5,6,7,8,9,10,11]. It is a new study filed, that is, how to organically combine nanoenzyme catalysis with its physical and chemical properties to create more novel functions. In analytical chemistry, nanoenzymes have been used in absorption, fluorescence, and chemiluminescence analysis [3,4,5,6,7,8,9,10,11,12,13]. Gold nanoparticles have peroxidase activity that catalyze the colored redox reaction of 3,3′,5,5′-tetramethylbenzidine (TMB)-H_2_O_2_ [12]. Combining glucose peroxidase and gold nanoenzyme, a 18–1100 μmol/L glucose can be detected spectrophotometrically, with a detection limit of 4 μmol/L. Li et al. [13] constructed CdTe dots and gold nanoparticles into the SiO_2_ microball surface respectively to obtain a nanoenzyme that catalytically oxidized to form oxidant, and a 1.32 μmol/L glucose can be determined by fluorescence method.

Mercury is a highly toxic heavy metal element which is very widely distributed in nature. Therefore, the monitoring of Hg^2+^ becomes very important. At present, several methods have been reported to determine Hg^2+^, such as atomic fluorescence spectroscopy (AFS), atomic absorption spectrometry (AAS), and inductively coupled plasma mass spectrometry (ICP-MS) [14,15,16]. Although these methods have high selectivity and sensitivity for Hg^2+^ detection, however, these methods have a number of weaknesses such as expensive instruments, long analysis period, need for professional operators, and so on. It is of great significance to develop a simple, rapid, and low-cost method for mercury detection. In recent years, some new methods for the detection of Hg^2+^ have been reported such as aptamer and functionalized gold nanoparticle biosensors [17,18,19]. Resonance Rayleigh scattering (RRS) method is a simple and sensitive spectral analysis technique. It has been used for the analysis of proteins, nucleic acids, metal ions, and so on [20,21,22,23,24]. Early studies of our group have developed sensitive and selective nanogold catalysis, immunonanogold catalysis, aptamer-nanogold catalysis, and peptide modified-nanogold catalysis methods [25,26,27,28,29,30,31], including the silver nitrate-hydroquinone [28] and HAuCl_4_-ascorbic acid reactions [29]. The above nanoenzyme catalytic RRS methods were all based on the aggregations of nanoparticles that presented unstable analytical systems and complicated operations. In this study, we have observed that AuNPs have strong catalysis on the redox reaction of HAuCl_4_-H_2_O_2_, while Hg^2+^ exhibits strong inhibition on this nanocatalytic reaction, and the nanoreaction product of formed AuNPs demonstrated RRS effect and SERS activity in the presence of Victoria blue B (VBB) probes. Thus, two simple, rapid, low cost, high sensitivity, and good selectivity RRS and SERS methods were established for detection of Hg^2+^.

## 2. Results and Discussion

### 2.1. Principle

**Figure 1:**
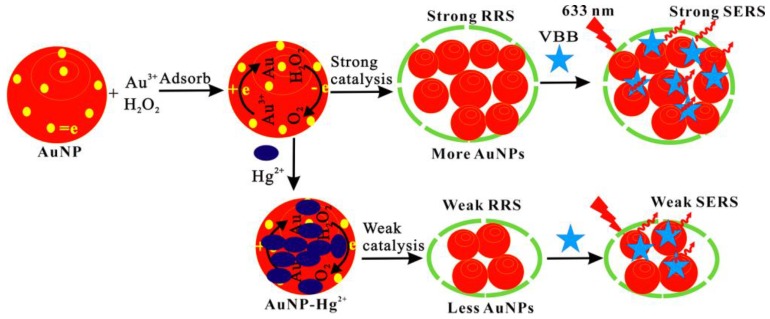
Resonance Rayleigh scattering (RRS)/surface-enhanced Raman scattering (SERS) detection of trace Hg^2+^ based on the inhibition of gold nanoparticles (AuNP) catalysis.

Nano-catalysis is an important way to amplify analysis signal. It has significance in developing a new nano-catalysis reaction. From the experiment, we found that, in hydrochloric acid medium, the reaction of H_2_O_2_-HAuCl_4_ was relatively slow. While nanocatalyst of AuNP was added, a large number of HAuCl_4_ adsorbed on the AuNP surface, and H_2_O_2_ was also adsorbed on the surface that there had many free electrons, which can enhance the redox electron-transfer between H_2_O_2_-HAuCl_4_. Thus, it enhanced the reduction of Au^3+^ to form elemental gold, and led to the gold nanoparticles forming greatly. The formed gold nanoparticles have strong SERS and RRS signal. Therefore, the nanogold catalytic reaction can be used to establish SERS and RRS detection methods for AuNPs (Figure 1).

Au^3+^ + AuNP = AuNP-Au^3+^ (adsorb)
H_2_O_2_ + AuNP-Au^3+^ = Au^3+^-AuNP-H_2_O_2_ (adsorb)
Au^3+^-AuNP-H_2_O_2_ = Au^+^-AuNP-H_2_O_2_ + O_2_
Au^+^-AuNP-H_2_O_2_ = AuNP + Au + O_2_
nAu = (Au)_n_ = AuNPs

It is known that Au and Hg have stronger affinities to form Au-amalgam than other metals such as Ag, Pb, and Cu. Thus, the stable AuNP-Hg^2+^ would form in the presence of Hg^2+^. Similarly, in the analytical system, the formed AuNP-HgCl_4_^2^^−^ can adsorb on the surface of AuNP, which greatly inhibits the red electron-transfer, and decreases the AuNP catalytic reaction. With the Hg^2+^ connection increased, the catalytic reaction rate slowed, and the RRS and SERS intensity decreased (Figure 1). Hereby, a RRS and SERS method was established for selective determination of Hg^2+^.

### 2.2. RRS Spectra

**Figure 2:**
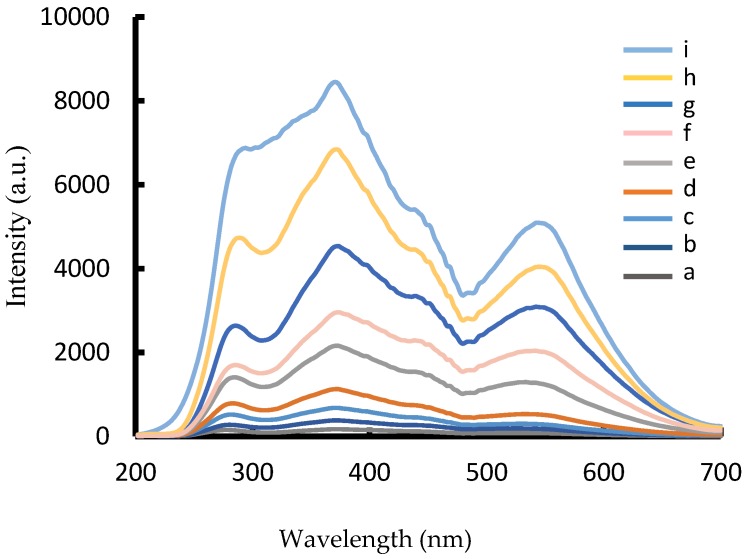
RRS spectra of the AuNP_b_-HAuCl_4_-H_2_O_2_ nanocatalytic system. (**a**) 4.48 µmol/L HAuCl_4_ + 0.67 mmol/L HCl + 3.33 mmol/L H_2_O_2_; (**b**) **a** + 0.018 ng/mL AuNP_b_; (**c**) **a** + 0.095 ng/mL AuNP_b_; (**d**) **a** + 1.9 ng/mL AuNP_b_; (**e**) **a** + 5.7 ng/mL AuNP_b_; (**f**) **a** + 7.6 ng/mL AuNP_b_; (**g**) **a** + 1.9 ng/mL AuNP_b_; (**h**) **a** + 5.7 ng/mL AuNP_b_; (**i**) **a** + 7.6 ng/mL AuNP_b_.

**Figure 3:**
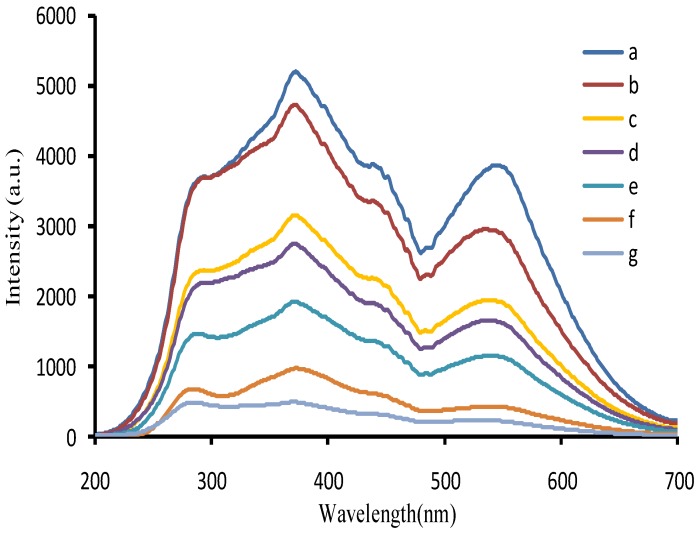
RRS spectra of the Hg^2+^-AuNP_c_-HAuCl_4_-H_2_O_2_ inhabited system. (**a**) 38 ng/mL AuNP_b_ + 4.48 µmol/L HAuCl_4_ + 0.67 mmol/L HCl + 3.33 mmol/L H_2_O_2_; (**b**) **a** + 0.08 µmol/L Hg^2+^; (**c**) **a** + 0.5 µmol/L Hg^2+^; (**d**) **a** + 0.67 µmol/L Hg^2+^; (**e**) **a** + 0.83 µmol/L Hg^2+^; (**f**) **a** + 1.17 µmol/L Hg^2+^; (**g**) **a** + 1.33 µmol/L Hg^2+^.

In HCl medium, nanocatalyst of AuNP_b_, AuNP_c_, and AgNP could catalyze H_2_O_2_ to reduced HAuCl_4_ to form AuNPs with three RRS peaks at 280, 370, and 550 nm. When nanocatalyst AuNP increased, the RRS intensity at 370 nm enhanced linearly (Figure 2 and Figure S1). The catalytic activity of AuNP_b_ was higher than AuNP_c_, due to the smaller particle size, and the larger specific surface area. When Hg^2+^ was adsorbed on the surface of AuNP_b_ to form AuNP_b_-HgCl_4_^2−^, the catalytic activity of AuNP_b_ was weakened. The RRS intensity at 370 nm decreased linearly with the Hg^2+^ concentration increased (Figure 3), and was selected for the determination of Hg(II). In addition, the RRS spectra of the Hg^2+^-AuNP_c_-HAuCl_4_-H_2_O_2_ system were recorded at room temperature. Results (Figure S2) show that the RRS signals were very weak and constant when Hg^2+^ concentration increased up to 12 µmol/L. This indicated that Hg^2+^ ions could strongly adsorb on the AuNP_b_ surfaces and there are no aggregations in the system.

### 2.3. SERS Spectra

**Figure 4:**
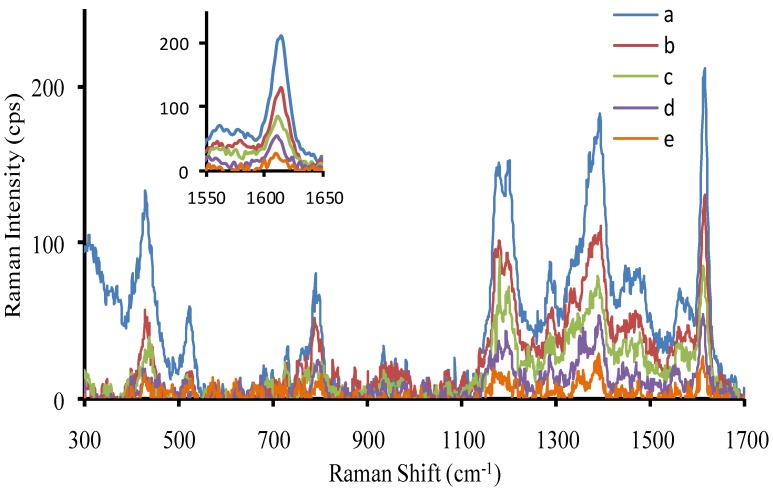
SERS spectra of the Hg^2+^-AuNP_b_-HAuCl_4_-H_2_O_2_-Victoria blue B (VBB) system. (**a**) 38 ng/mL AuNP_b_ + 4.48 µmol/L HAuCl_4_ + 0.67 mmol/L HCl + 3.33 mmol/L H_2_O_2_−1.3 µmol/L VBB; (**b**) **a** + 0.013 µmol/L Hg^2+^; (**c**) **a** + 0.17 µmol/L Hg^2+^; (**d**) **a** + 0.33 µmol/L Hg^2+^; (**e**) **a** + 0.5 µmol/L Hg^2+^.

SERS is a sensitive molecular spectral technique [31,32,33], and has been used in trace analysis. Recently, it was selected to study some nanocatalysis reactions [32,33], with good results. In this article, it was used to examine the Hg(II)-AuNP_b_-H_2_O_2_-HAuCl_4_-molecular probe system. When RhS was used as SERS probe, it could adsorb on the generated gold nanoparticle surfaces that exhibited strong SERS peaks at the Raman shifts of 618, 732, 1199, 1277, 1356, 1507, 1527, and 1645 cm^−1^. Among them, the SERS peak at 1645 cm^−^^1^ was most sensitive and was selected for use. With the AuNP_b_ increase, the intensity of SERS at 1645 cm^−^^1^ increased linearly (Figure S3). Using VBB as a SERS probe, it displayed SERS peaks at the Raman shifts of 795, 1167, 1200, 1364, 1394, and 1612 cm^−1^. Among them, the SERS peak at 1612 cm^−1^ was the most sensitive. As the AuNP_b_ increased, the intensity of SERS at 1612 cm^−1^ linearly increased (Figure S4). Using safranin T as SERS probe, it displayed SERS peaks at the Raman shifts of 349, 612, 1240, 1372, 1551, and 1639 cm^−1^. Among them, the SERS peak at 1372 cm^−1^ was the most sensitive. With the AuNP_b_ increased, the intensity of SERS at 1372 cm^−1^ linearly increased (Figure S5). Rhodamine 6G was tested as a SERS probe, but its Raman intensity was very weak. When Hg^2+^ adsorbed on the surface of AuNP_b_, the nanocatalytic activity weakened, the peak at 1612 cm^−1^ decreased linearly, and the AuNP_b_ catalytic system was chosen for quantitative analysis of Hg(II) (Figure 4).

### 2.4. Absorption Spectra

AuNP_b_ exhibited a surface plasmon resonance (SPR) peak at 510 nm, it catalyzed H_2_O_2_ reduced HAuCl_4_ to form gold nanoparticles with a SPR peak at 570 nm. With AuNP_b_ increased, the color changed from colorless to red (Figure S6) and the SPR peak value *A_570 n_*_m_ increased (Figure S7). When AuNP_c_ was used as nanocatalyst, it had a SPR peak at 590 nm. As AuNP_c_ increased, the SPR peak increased (Figure S8). AgNP exhibited the nanocatalysis on the reaction, the SPR peak increased with the nanocatalyst increase (Figure S9). However, when Hg^2+^ was added to the AuNP_b_, the intensity at 560 nm of the nanocatalytic system decreased linearly (Figure S10), and the peak was chosen for the detection of Hg, with lowest-cost. Furthermore, it can be detected by lake-eye.

### 2.5. Scanning Electron Microscopy (SEM)

**Figure 5:**
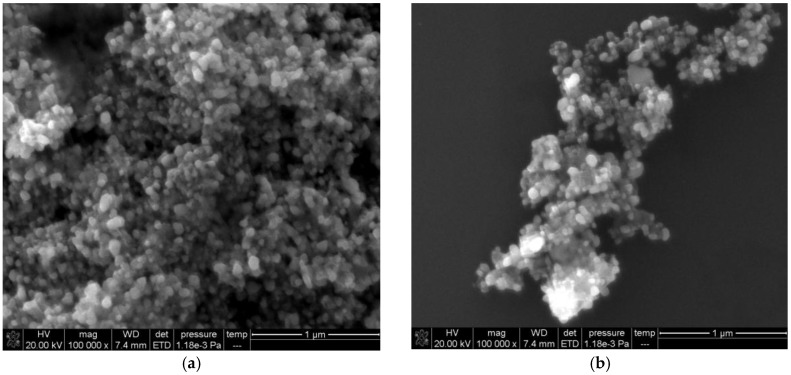
Scanning Electron Microscopy (SEM) images of the AuNP_b_ catalytic system. (**a**) 0.67 mmol/L HCl + 4.48 µmol/L HAuCl_4_ + 3.33 mmol/L H_2_O_2_ + 19 ng/mL AuNP_b_; (**b**) **a** + 1 µmol/L Hg^2+^.

The AuNP_b_, AuNP_c_, and Ag NPs were in spherical shape in size of 5, 10, and 9 nm respectively (Figure S11). For the HAuCl_4_-H_2_O_2_ system, the reaction rate was slow in the absence of AuNP_b_ and there were few gold nanoparticles in reaction solution. When nanocatalyst of AuNP_b_ was added, a plenty of irregular gold nanoparticles were generated (Figure 5a). As Hg^2+^ was added, it inhibited the nanocatalytic reaction of AuNP_b_-H_2_O_2_-HAuCl_4_. The generated gold nanoparticles were reduced (Figure 5b).

### 2.6. Optimization of Analytical Conditions

The effect of HCl concentration on the AuNP_b_-H_2_O_2_-HAuCl_4_ catalytic reaction was considered. The amount of HCl had great influences on the generated nanogold. When the concentration was 0.67 mmol/L, the *ΔI* value was the largest, and the color was pink with *I_370 nm_* of 3506, and the blank was colorless with *I_370 nm_* of 506. When HCl concentration continued to increase, a large number of hydrogen ions in the solution limited the redox of H_2_O_2_-HAuCl_4_. Therefore, the concentration of 0.67 mmol/L HCl was selected in this experiment (Figure S12). We also considered the effect of the HAuCl_4_ concentration on the catalytic reaction, and found that the *ΔI* value was the largest when the concentration was 4.48 µmol/L (Figure S13). When HAuCl_4_ concentration increased continuously the SERS value held constant due to forming the largest AuNPs. The effect of H_2_O_2_ concentration on *ΔI* was studied and the best concentration was 3.33 mmol/L (Figure S14). The effect of the temperature on the catalytic reaction was investigated. The *ΔI* value increased with a temperature increase in the range of 30–60 °C due to an increase in the number of formed AuNPs. When the temperature was 60 °C, the *ΔI* value was the largest due to formed largest AuNPs, and the blank formed small nanogolds with a pale pink color. When reaction temperature was higher than 60 °C, the *ΔI* value decreased due to the blank increasing significantly. Thus, 60 °C was chosen (Figure S15). We also investigated the heating time and 15 min was selected (Figure S16). We found that when the heating time exceeded 15 min, the blank generated nanogolds and the RRS intensity increased. Even when the heating time was 25 min, the blank solution presented a reddish color that meant a large amount of nanogold was produced. Moreover, after heating, the system was immediately cooled in an ice bath to stop the reaction. Effect of RhS, VBB, and safranine T concentration on the SERS intensity was investigated, and 7, 13.2, and 6.7 µmol/L were selected respectively (Figures S17–S19).

### 2.7. Effect of Foreign Substances

According to the procedure, the effect of foreign substances on the determination of 1.5 × 10^−7^ mol/L Hg^2+^ was tested, when the relative error was within ±10%. The results indicated that 8.3 × 10^−5^ mol/L Cu^2+^; 3.3 × 10^−5^ mol/L I^−^ and Cd^2+^; 6.7 × 10^−6^ mol/L Zn^2+^ and Bi^3+^; 3.3 × 10^−6^ mol/L Pb^2+^; 2.0 × 10^−^^6^ mol/L S^2−^; Pd^2+^ and Pt^2+^; 1.7 × 10^−6^ mol/L Co^2+^; and Cr^3+^ and Ni^2+^ did not interfere with the determination. It indicated that this method had a good selectivity due to stronger intermolecular forces between Au and Hg than the other Au metals.

### 2.8. Working Curve

Under optimal conditions, the relationship between the nanocatalyst concentration of AuNP_b_, AuNP_c_, and AgNPs and their corresponding Δ*I_370 nm_* were obtained (Table 1, Figures S20–S22). The results showed that the AuNP_b_ is the strongest catalyst with maximum slope, and it was chosen for use. For the AuNP_b_-HAuCl_4_-H_2_O_2_ nanocatalytic system, using RhS, VBB, and safranine T as molecular probes respectively, their SERS intensities were recorded. The experimental results indicated that VBB was the most sensitive SERS probe (Figures S23–S25). For the Hg^2+^ analytical system, the RRS, SERS, and Abs methods were studied (Figures S26–S28). From Table 1, we can see that the RRS is most sensitive and was chosen for detection of Hg. The RRS method was compared with the reported methods for determination of Hg^2+^ [34,35,36,37], this method is more simple, and the reagent is very easy to obtain, with high sensitivity and good selectivity.

### 2.9. Analysis of Samples

Three water samples—including tap, river, and pond—were collected from Guangxi Normal University, and filtrated according to the reference [35]. The following operations were according to the procedure to detect Hg^2+^. The obtained results were accorded with the cold atomic absorption spectrometry (AAS) (Table S1). The average values (*n* = 5) for the water samples after adding a certain amount of Hg^2+^ were determined. The recovery was in the range of 97–102%, and the relative standard deviation (RSD) was in the range of 4.5–5.2%. This indicated the method is accuracy and reliable.

## 3. Materials and Methods

### 3.1. Apparatus

A DXR smart Raman spectrophotometer ( Thermo Fisher, Waltham, MA, USA) with a power of 2.5 mW, laser wavelength of 633 nm, and slit width of 50 mm; a Cary Eclipse fluorescence spectrophotometer (Varian, Santa Clara, CA, USA); a model of TU-1901 double-beam UV—visible spectrophotometer (Beijing Purkinje General Instrument Co., Ltd., Beijing, China); a C-MAG HS7 magnetic stirrer with heating (IKA, Staufen, Germany), and a constant temperature magnetic stirrer(Kewei Yongxing Instrument Co., Ltd., Beijing, China) were used. A FEI Quanta 200 FEG Field emission scanning electron microscope (FEI, Hillsboro, OR, USA) was recorded the SEM of nanoparticles, the sample preparation was as follows: put 1.5 mL of the reaction solution of the procedure into a 2 mL centrifuge tube and centrifuge for 20 min (150 × 100 r/min), discard the supernatant and add water to 1.5 mL, then dispers for 30 min with ultrasonication. After centrifuging again, add 1 mL water, and disperse for 30 min. Place 2 µL of the sample solution by pipette dripping on silicon slices and dry naturally for use.

### 3.2. Reagents

A 1% HAuCl_4_·4H_2_O (National Medicine Group Chemical Reagent Co., Ltd., Shanghai, China), 1% trisodium citrate, 10 mmol/L sodium borohydride, 0.01 mol/L HCl solution and 0.3% H_2_O_2_ (0.1 mol/L) were prepared. Victoria blue B solution: 0.025 g VBB was dissolved in 5.0 mL ethyl alcohol before diluting to 50.0 mL with water, and the concentration was 1.0 × 10^−3^ mol/L. It was stepwise diluted to 1.0 × 10^−5^ mol/L before use. A 5.23 × 10^−5^ mol/L RhS and 5.0 × 10^−5^ mol/L safranine T solution were prepared. Preparation of gold nano sol (AuNP_b_): At room temperature, 40 mL of water was added into a conical flask, 0.5 mL 1.0% HAuCl_4_ and 3.5 mL 1.0% trisodium citrate were added into the flask in order by stirring. Then, 4.0 mL 0.05% NaBH_4_ was added slowly. The mixture was stirred for 10 min, to obtain a concentration of 58 μg/mL 5 nm AuNP_b_ . Preparation of AuNP_c_: 50 mL of water was added into a conical flask, heated to boiling. Then, 0.5 mL 1% HAuCl_4_ and 3.5 mL 1% trisodium citrate were added rapidly into the boiling water successively. After boiling for 10 min while stirring, the color went from colorless to wine red. The mixture was stirred continuously to room temperature, and then diluted to 50.0 mL to obtain about 10 nm of AuNP_c_ at a concentration of 58 μg/mL. Preparation of silver nanosol (AgNPs): 40 mL of water was added into a conical flask, 385 μL 2.4 × 10^−2^ mol/L AgNO_3_ and 3.5 mL 10 g/L trisodium citrate were added into the flask in order with stirring. Then, 4.0 mL 0.5 mg/mL NaBH_4_ was added slowly. The color turned from pale yellow to deep yellow. The mixture was stirred for 10 min, diluted to 50.0 mL to obtain a concentration of 20.0 μg/mL AgNPs about 9 nm in size, and stored at 4 °C. All reagents were of analytical grade and the water was doubly distilled.

### 3.3. Procedure

In a 5 mL marked test tube, 100 µL 0.57 µg/mL AuNP_b_, and a certain amount of Hg^2+^ were added and diluted to 200 µL, then left to rest for about 20 min. 80 µL 0.1% HAuCl4 (84 μmol/L), 100 μL 0.01 mol/L HCl, and 50 µL 0.3% (0.1 mol/L) H_2_O_2_ were added sequentially, diluted to 1.5 mL and mixed well. Then the mixture heated for 15 min in a 60 °C water bath and cooled with tap water. The RRS spectra were recorded by means of synchronous scanning excited wavelength *λ_ex_* and emission wavelength *λ_em_* (*λ_ex_* − *λ_em_* = *Δλ* = 0) on fluorescence spectrophotometer, with a photomultiplier tube (PMT) voltage of 400 v, and both excited and emission slit widths were 5 nm, emission filter = 1% T attenuator. The reaction solution RRS intensity at 370 nm *I_370 nm_* and the blank solution without Hg^2+^ (*I_370 nm_*)_0_ were recorded. The value of *ΔI_370 nm_* = *I_370 nm_* − *(I_370 nm_)_0_* was calculated. For the SERS detection, a 200 μL 1.0 × 10^−5^ mol/L VBB was added in the reaction solution, mixed well, and transferred to a 1 cm quartz cell. Its SERS spectra were recorded by the Raman spectrophotometer. The SERS intensity at 1612cm^−1^ (*I*) and the blank value (*I_0_*) without Hg^2+^ were recorded. The value of *ΔI* = *I* − *I_0_* was calculated.

## 4. Conclusions

The gold nanoreaction of HAuCl_4_-H_2_O_2_ is slow. Upon addition of nanocatalyst of AuNP_b_, the nanoreaction enhanced greatly to form gold nanoparticles with strong RRS, SERS and SPR absorption effects. When analyte of Hg^2+^ was added, the catalysis was greatly inhibited and the SPR effect decreased. Thus, two new RRS and SERS methods were developed for determination of Hg^2+^, with simplicity, high sensitivity, and good selectivity.

## Figures and Tables

**Table 1:** Analytical features of the nanocatalytic analytical systems.

Analyte	Method	Regression Equation	Linear Range (µmol/L)	Coefficient	Detection Limit (µmol/L)
AuNP_b_	RRS	Δ*I_370 nm_* = 131.3*C* + 300	0.025–25	0.9951	0.008
AuNP_c_,	RRS	Δ*I_370 nm_* = 51.5*C* + 267	0.05–75	0.9941	0.02
AgNP	RRS	Δ*I_370 nm_* = 23.4*C* + 73	0.5–50	0.9971	0.2
AuNP_b_	SERS ^a^	Δ*I_1645 cm^−1^_* = 2.28*C* + 72	0.5–50	0.9786	0.2
AuNP_b_	SERS ^b^	Δ*I_1612 cm^−1^_* = 5.94*C* + 86	0.2–50	0.9942	0.1
AuNP_b_	SERS ^c^	Δ*I_1372 cm^−1^_* = 1.47*C* − 9.1	0.6–50	0.9879	0.3
Hg^2+^	RRS	Δ*I_370 nm_* = 3650*C* + 111	0.008–1.33	0.9958	0.003
Hg^2+^	SERS ^b^	Δ*I_1612 cm^−1^_* = 326*C* + 6.4	0.013–0.5	0.9932	0.03
Hg^2+^	Abs	ΔA_600 nm_ = 0.083*C* + 0.0087	0.5–2.33	0.9876	0.2

^a^ RhS; ^b^ VBB; ^c^ safranine T.

## Supplementary Materials

The following are available online at http://www.mdpi.com/2079-4991/7/5/114/s1.
