# Supplementary material for: Resonance Rayleigh Scattering and SERS Spectral Detection of Trace Hg(II) Based on the Gold Nanocatalysis
Source: Nanomaterials (Basel). 2017 May 17;7(5):114. doi: 10.3390/nano7050114 (PMC5449995; doi:10.3390/nano7050114)
Supplement: Supplementary file 1 [file nanomaterials-07-00114-s001.pdf]

Resonance Rayleigh Scattering and SERS Spectral Detection of Trace Hg(II) Based on the Gold Nanocatalysis

Huixiang Ouyang[1,2], Chongning Li[1], Qinye Liu[1], Guiqing Wen[1], Aihui Liang[1*], Zhiliang Jiang[1*]

[1]Key Laboratory of Ecology of Rare and Endangered Species and Environmental Protection of Ministry Education, Guangxi Key Laboratory of Environmental Pollution Control Theory and Technology, Guangxi Normal University, Guilin 541004, China; [2] Guangxi Colleges and Universities Key Laboratory of Regional Ecological Environment Analysis and Pollution Control of West Guangxi, College of Chemistry and Enviroment Engineering, Baise University, Baise 533000, China

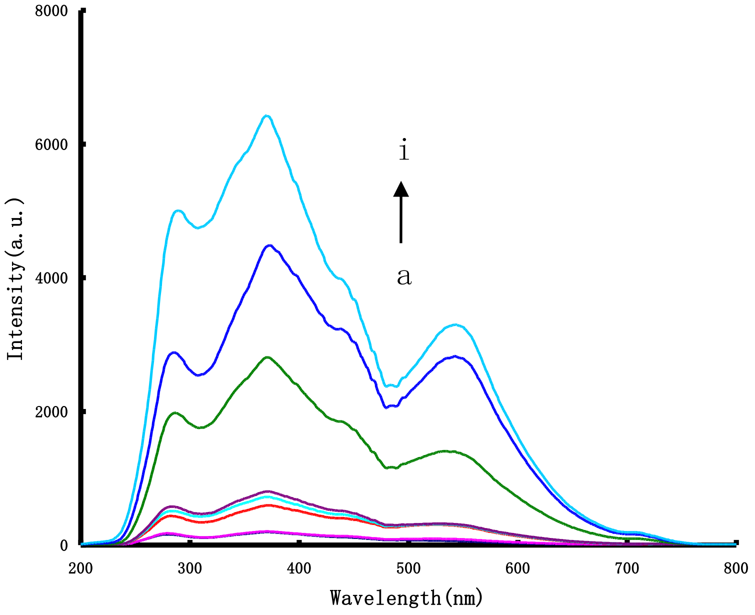

Figure S1 RRS spectra of the AgNPs-$HAuCl_4$-H_2O_2 nanocatalytic system

(a)4.48µmol/L $HAuCl_4$+0.67mmol/L HCl+3.33mmol/L H_2O_2; (b)a+3.3ng/mL AgNPs; (c)a+6.6ng/mL AgNPs; (d)a+13.3ng/mL AgNPs;(e)a+33.2 ng/mL AgNPs; (f)a+99.5 ng/mL AgNPs;（g）a+133ng/mL AgNPs;（h） a+57ng/mL AgNPs;（i） a+265ng/mL AgNPs.

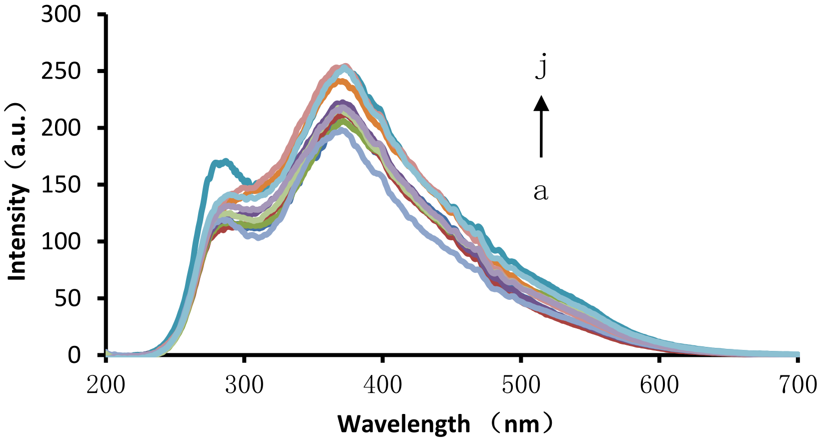

Figure S2 RRS spectra of the Hg^{2+}-$AuNP_c$-$HAuCl_4$-H_2O_2 system

(a)38ng/mL $AuNP_b$ +4.48μmol/L $HAuCl_4$+0.67mmol/L HCl+3.33mmol/L H_2O_2, 15 min at room temperature; (b)a+0.013μmol/L Hg^{2+}; (c)a+0.17μmol/L Hg^{2+}; (d)a+0.67μmol/L Hg^{2+}; (e)a+0.83μmol/L Hg^{2+}; (f)a+1.17μmol/L Hg^{2+};(g)a+1.33μmol/L Hg^{2+};(h)a+3 μmol/L Hg^{2+}; (i)a+6 μmol/L Hg^{2+}; (j)a+12 μmol/L Hg^{2+}.

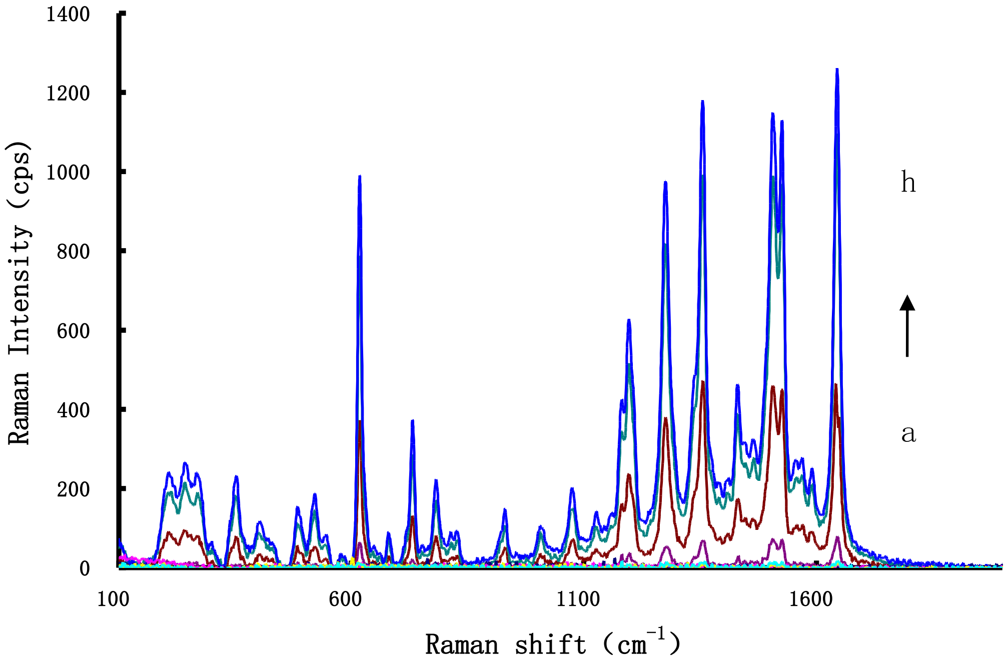

Figure S3 SERS spectra of the $AuNP_b$-$HAuCl_4$-H_2O_2-RhS system

（a）4.48μmol/L $HAuCl_4$+0.67mmol/L HCl+3.33mmol/L H_2O_2+0.70μmol/L RhS（b）a+3.8ng/mL $AuNP_b$;（c）a+19ng/mL $AuNP_b$; （d）a+38ng/mL $AuNP_b$; （e）a+95ng/mL $AuNP_b$;（f）a+190 ng/mL $AuNP_b$; （g）a+380 ng/mL $AuNP_b$;（h）a+456 ng/mL $AuNP_b$;

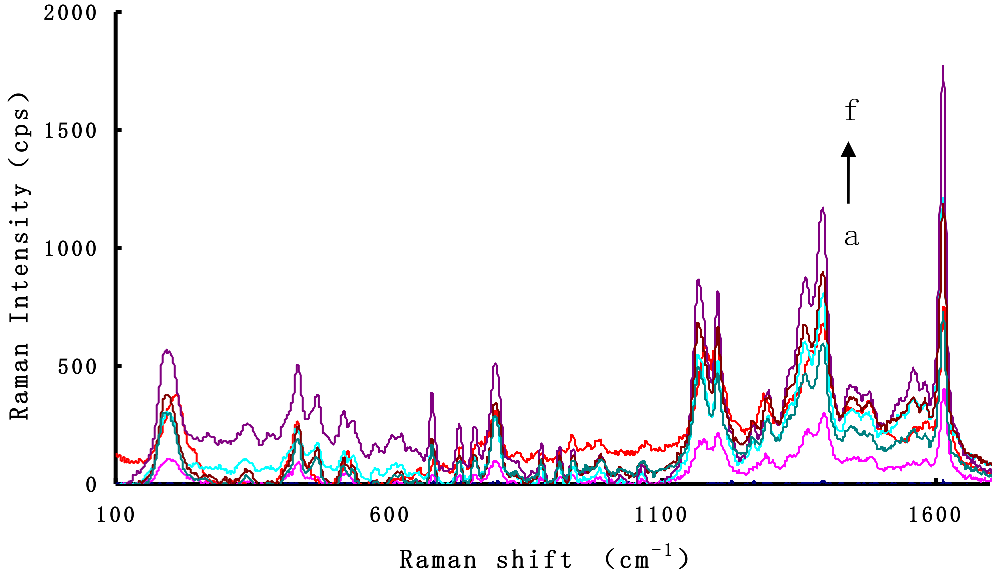

Figure S4 SERS spectra of the $AuNP_b$-$HAuCl_4$-H_2O_2-VBB system

（a）4.48μmol/L $HAuCl_4$+0.67mmol/L HCl+3.33mmol/LH_2O_2 +1.3μmol/L VBB（b）a+19ng/mL $AuNP_b$;（c）a+38ng/mL $AuNP_b$;（d）a+95ng/mL $AuNP_b$;（e）a+190 ng/mL $AuNP_b$;（f）a+285 ng/mL $AuNP_b$;

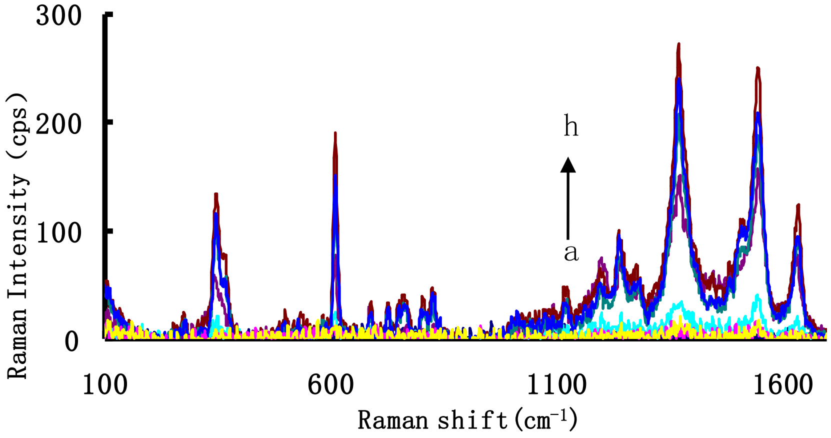

Figure S5 SERS spectra of the $AuNP_b$-$HAuCl_4$-H_2O_2- Safranin T system

（a）4.48μmol/L $HAuCl_4$+0.67mmol/L HCl+3.33mmol/L H_2O_2 +6.7mmol/L Safranin T（b）a+3.8ng/mL $AuNP_b$;（c）a+19ng/mL $AuNP_b$;（d）a+38ng/mL $AuNP_b$;（e）a+95ng/mL $AuNP_b$;（f）a+190 ng/mL $AuNP_b$;（g）a+295 ng/mL $AuNP_b$;（h）a+380 ng/mL $AuNP_b$;

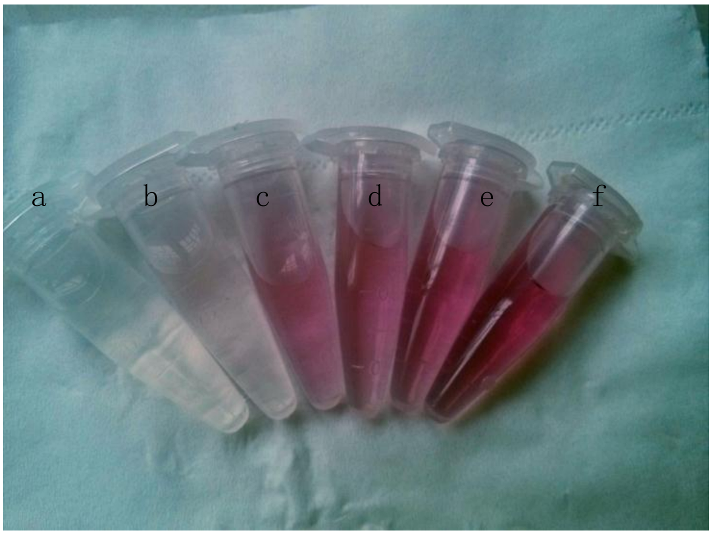

Figure S6 The color change of the $AuNP_b$-$HAuCl_4$-H_2O_2 system

（a）4.48μmol/L $HAuCl_4$+0.67mmol/L HCl+3.33mmol/L H_2O_2;（b）a+19 ng/mL $AuNP_b$; （c）a+95 ng/mL $AuNP_b$;（d） a+285ng/mL $AuNP_b$;（e） a+380 ng/mL $AuNP_b$; （f） a+760 ng/mL $AuNP_b$.

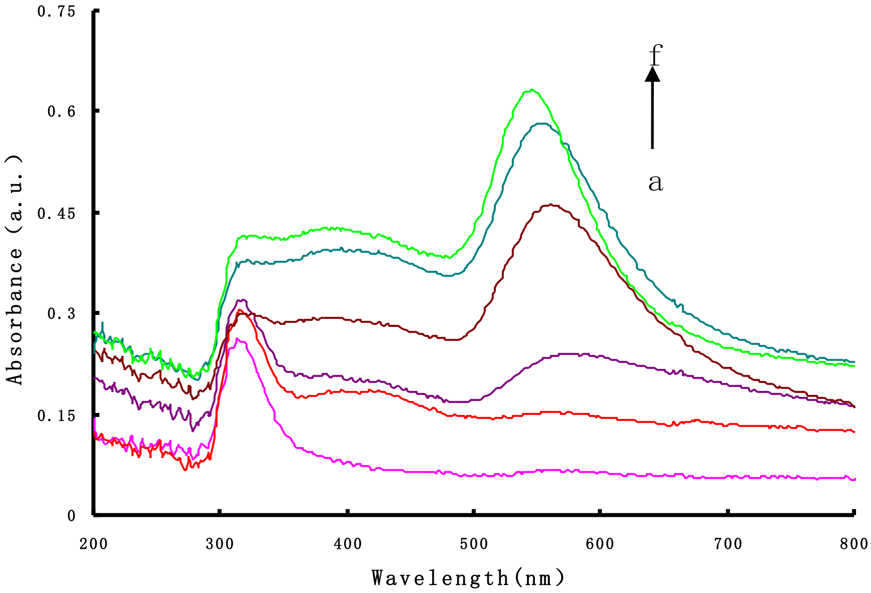

Figure S7 Absorption spectra of the $AuNP_b$-$HAuCl_4$-H_2O_2 system

（a） 4.48μmol/L $HAuCl_4$+0.67mmol/L HCl+3.33mmol/L H_2O_2 （b） a+9.5ng/mL $AuNP_b$; （c） a+38ng/mL $AuNP_b$;（d）a+133ng/mL $AuNP_b$;（e）a+190 ng/mL $AuNP_b$;（f）a+380ng/mL $AuNP_b$.

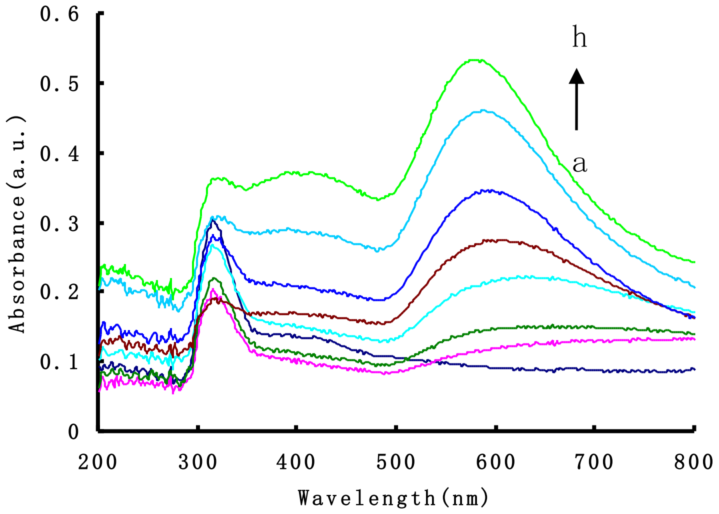

Figure S8 Absorption spectra of the AuNP$_c$-HAuCl$_4$-H$_2$O$_2$ system

（a）4.48μmol/L HAuCl$_4$+0.67mmol/L HCl+3.33mmol/L H$_2$O$_2$ （b）a+38ng/mL AuNP$_c$; （c）a+57ng/mL AuNP$_c$;（d）a+85.5ng/mL AuNP$_c$;（e）a+133 ng/mL AuNP$_c$;（f）a+152ng/mL AuNP$_c$;（g）a+190ng/mL AuNP$_c$;（h）a+228ng/mL AuNP$_c$

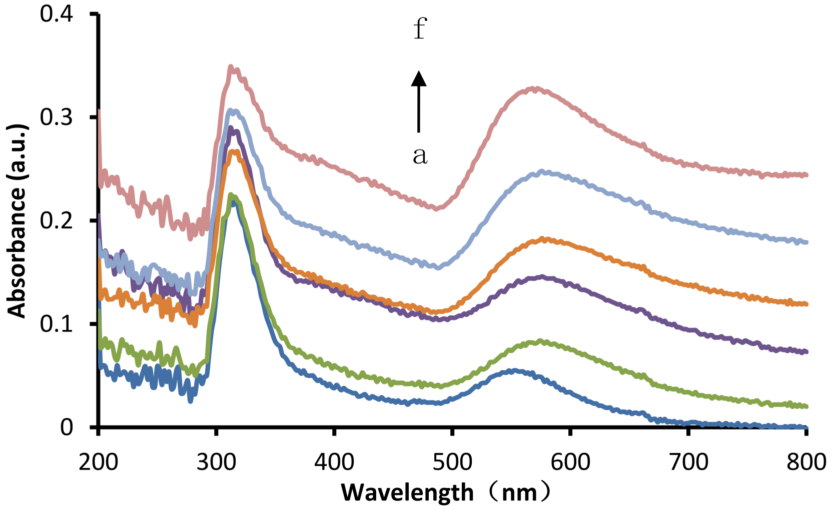

Figure S9 Absorption spectra of the AgNP$_s$-HAuCl$_4$-H$_2$O$_2$ system

（a）4.48μmol/L HAuCl$_4$+0.67mmol/L HCl+3.33mmol/L H$_2$O$_2$ （b）a+13ng/mL AgNPs; （c）a+60ng/mL AgNPs;（d)a+100ng/mL AgNPs;（e)a+166 ng/mL AgNPs;（f)a+265 ng/mL AgNPs;

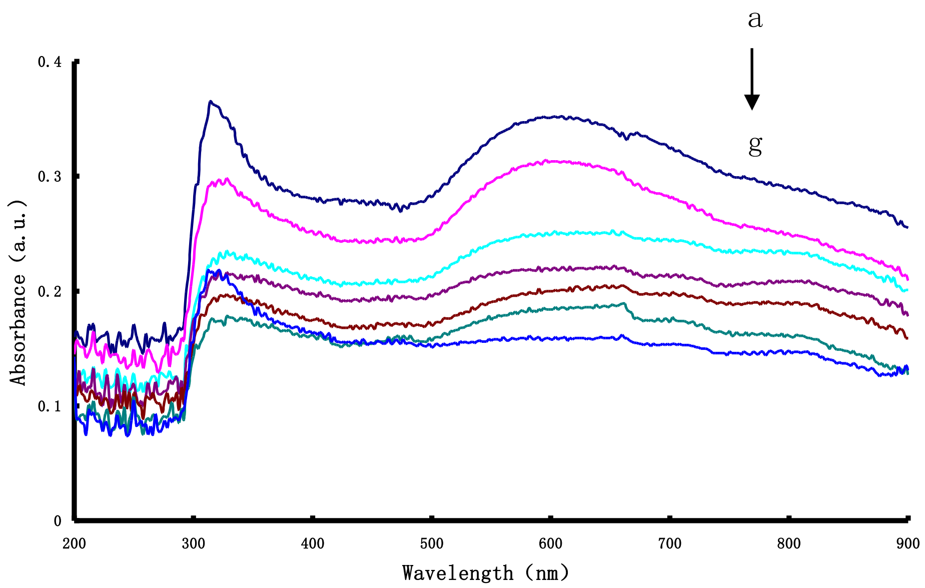

Figure S10 Absorption spectra of the Hg^{2+}-$AuNP_c$-$HAuCl_4$-H_2O_2 system

(a) 38ng/mLAuNP$_c$ +4.48μmol/L $HAuCl_4$+0.67mmol/L HCl+3.33mmol/L H_2O_2 (b) a+0.5μmol/L Hg^{2+}; (c) a+0.83μmol/L Hg^{2+}; (d) a+1.00μmol/L Hg^{2+}; (e) a+1.33μmol/L Hg^{2+}; (f) a+2.00μmol/L Hg^{2+}; (g) a+2.33μmol/L Hg^{2+}; (h) a+2.67μmol/L Hg^{2+}.

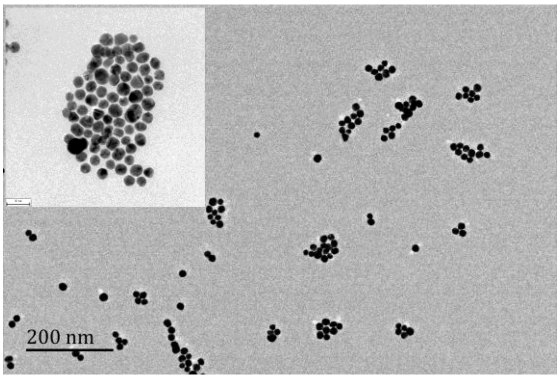

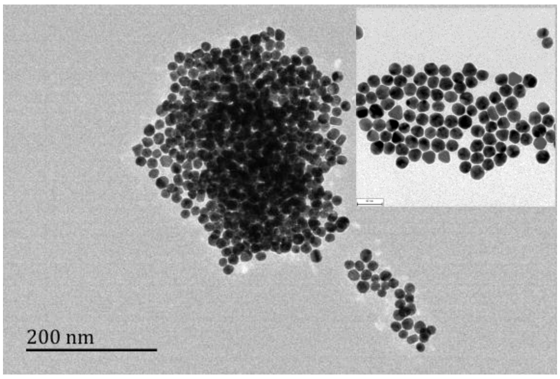

a b

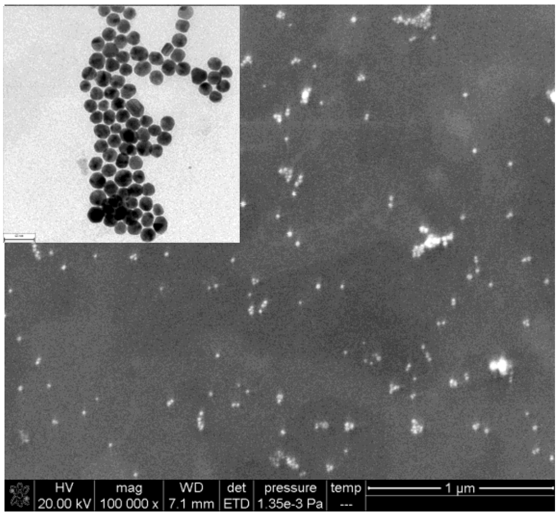

C

Figure S11. The TEM images for $AuNP_b$(a), $AuNP_c$(b) and Ag NPs (c).

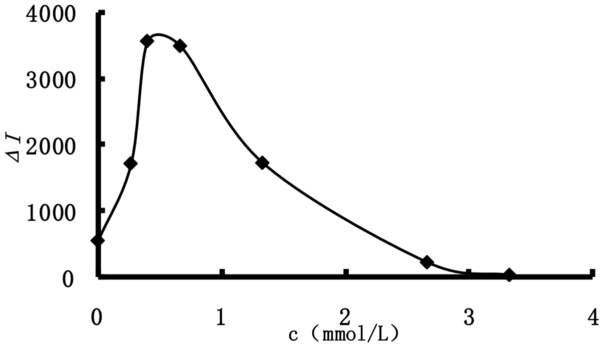

Figure S12 Effect of HCl concentration

2.24μmol/L $HAuCl_4$- HCl-3.33mmol/L H_2O_2 -152ng/mL $AuNP_b$;

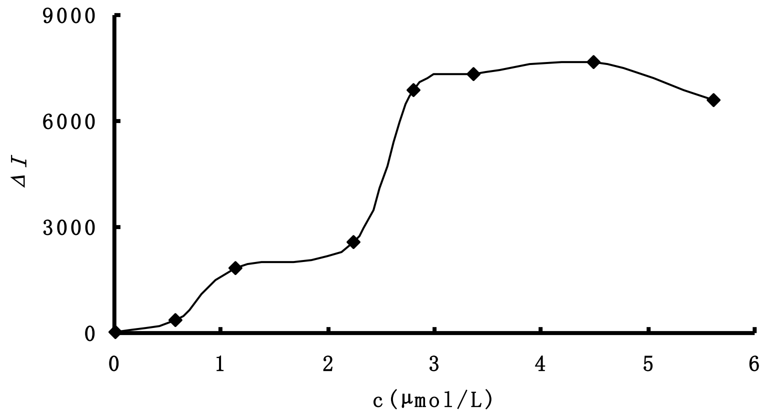

Figure S13 Effcet of $HAuCl_4$ concentration

$HAuCl_4$- 0.67mmol/L HCl-3.33mmol/L H_2O_2-152ng/mL $AuNP_b$.

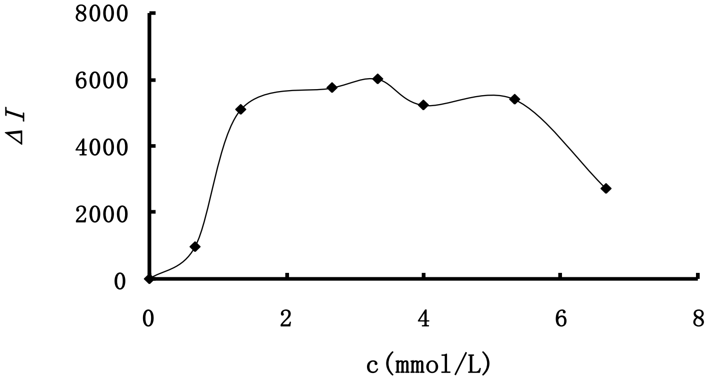

Figure S14 Effcet of H_2O_2 concentration

4.48μmol/L $HAuCl_4$- 0.67mmol/L HCl-H_2O_2-152ng/mL $AuNP_b$.

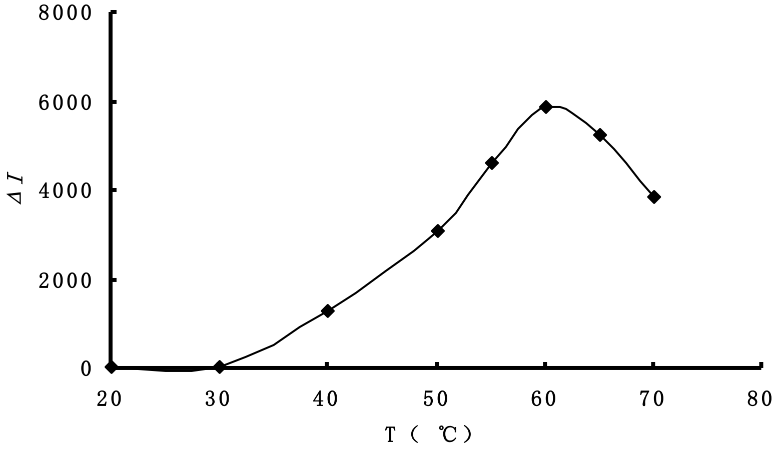

Figure S15 Effcet of temperature

4.48μmol/L $HAuCl_4$- 0.67mmol/L HCl-3.33mmol/L H_2O_2 -152ng/mL $AuNP_b$;

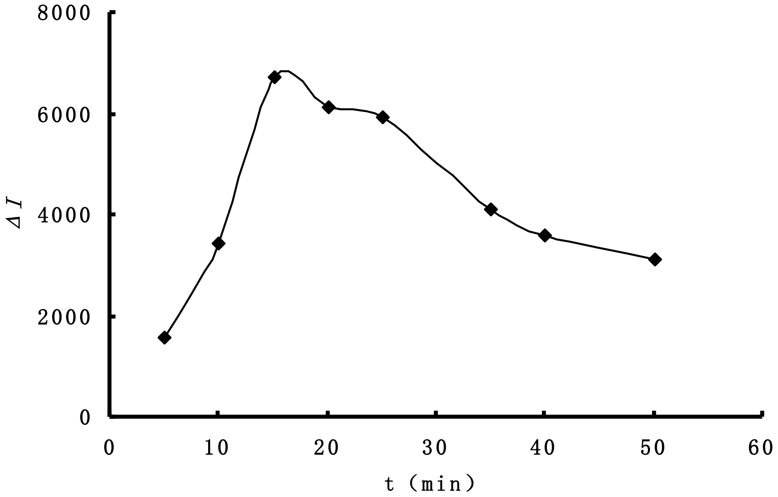

Figure **S16** Effcet of heating time

4.48μmol/L $HAuCl_4$- 0.67mmol/L HCl-3.33mmol/L H_2O_2 -152ng/mL $AuNP_b$;

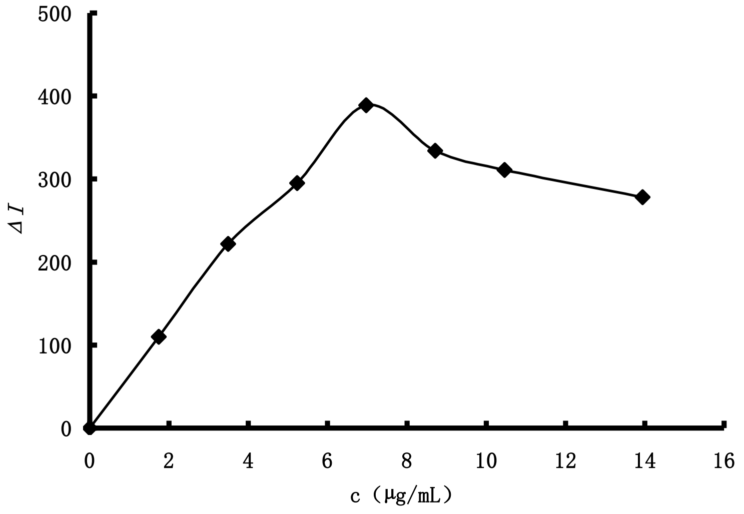

Figure S17 Effect of RhS SERS probe concentration

4.48μmol/L $HAuCl_4$- 0.67mmol/L HCl-3.33mmol/L H_2O_2 -190ng/mL $AuNP_b$-RhS;

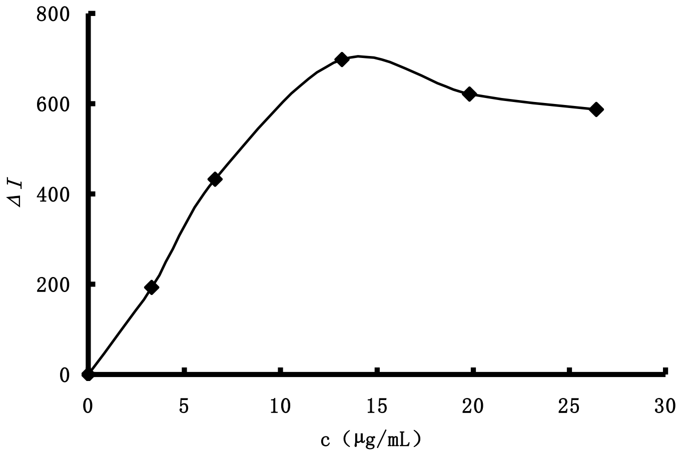

Figure S18 Effect of VBB SERS probe concentration

4.48μmol/L $HAuCl_4$- 0.67mmol/L HCl-3.33mmol/L H_2O_2 -190ng/mL $AuNP_b$-VBB;

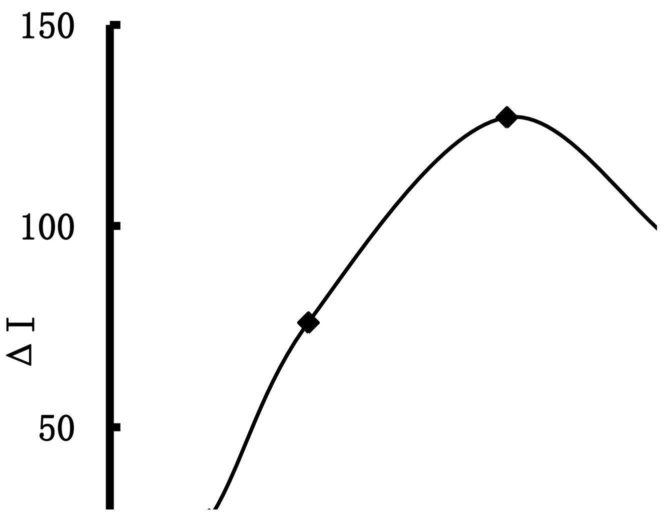

Figure S19 Effect of safranine T SERS probe concentration.

4.48μmol/L $HAuCl_4$- 0.67mmol/L HCl-3.33mmol/L H_2O_2 -190ng/mL $AuNP_b$- safranine T.

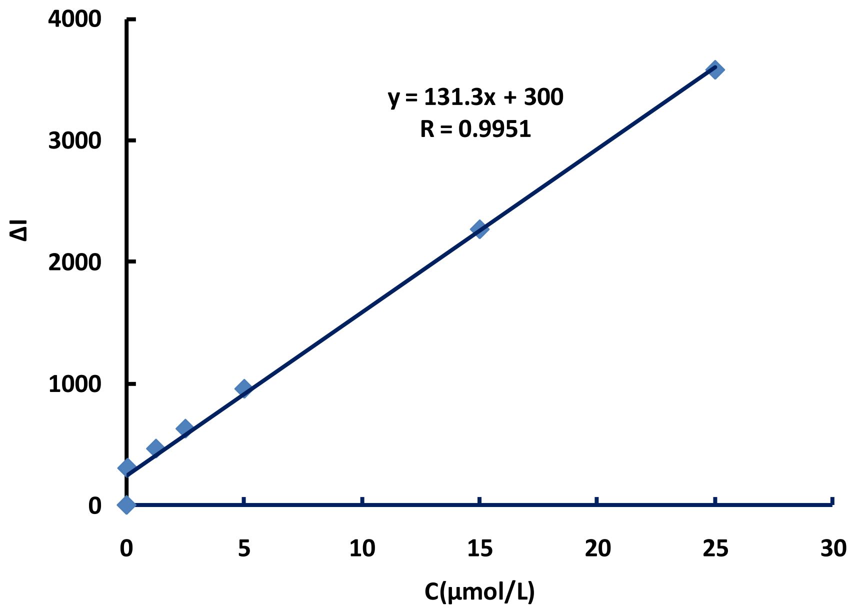

Figure S20 Working curve for RRS detection of $AuNP_b$
4.48μmol/L $HAuCl_4$-0.67mmol/L HCl-3.33mmol/L H_2O_2-$AuNP_b$.

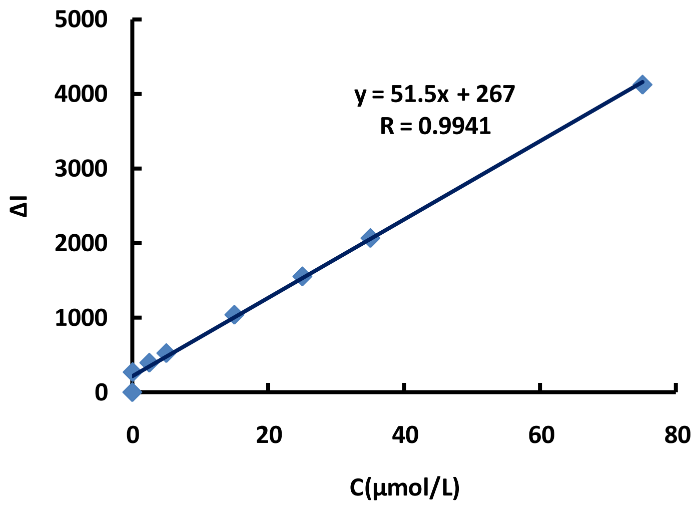

Figure S21 Working curve for SERS detection of AuNPc
4.48μmol/L $HAuCl_4$-0.67mmol/L HCl-3.33mmol/L H_2O_2-AuNPc.

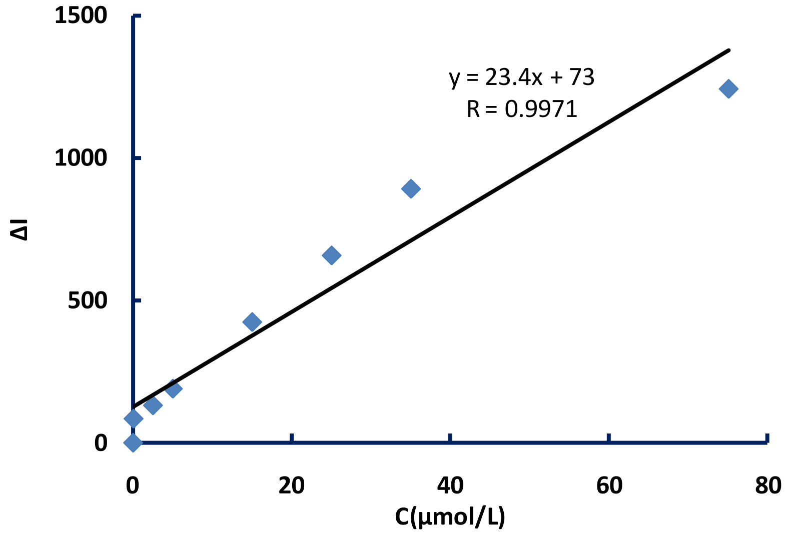

Figure S22 Working curve for SERS detection of AgNP
4.48μmol/L $HAuCl_4$-0.67mmol/L HCl-3.33mmol/L H_2O_2-AgNP.

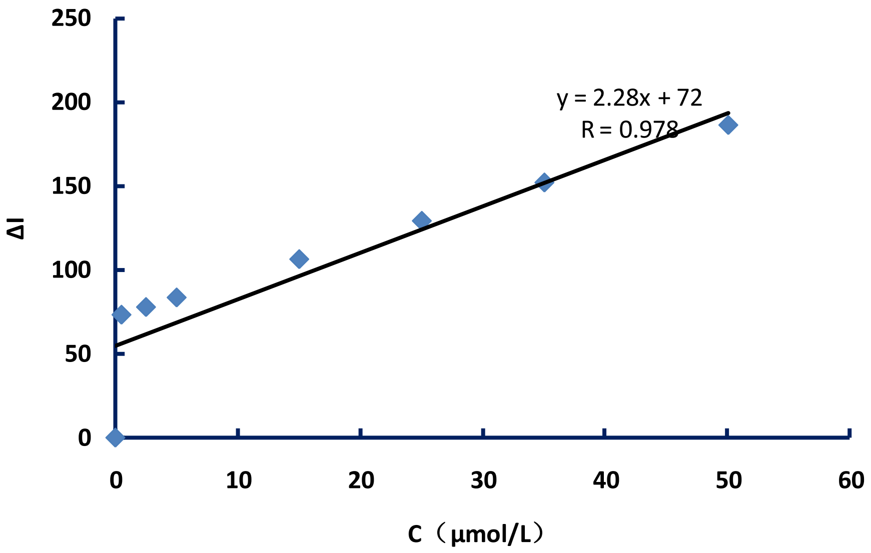

Figure S23 Working curve for SERS detection of $AuNP_b$ with RhS probe
4.48μmol/L $HAuCl_4$-0.67mmol/L HCl-3.33mmol/L H_2O_2-$AuNP_B$-6.97μmol/L RhS.

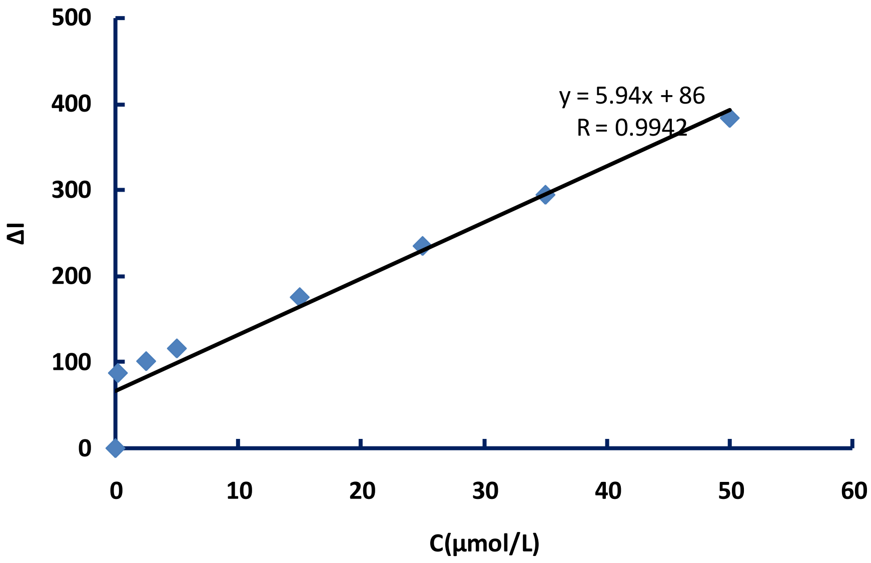

Figure S24 Working curve for SERS detection of $AuNP_b$ with VBB probe

4.48μmol/L $HAuCl_4$-0.67mmol/L HCl-3.33mmol/L H_2O_2-$AuNP_B$-1.3μmol/L VBB

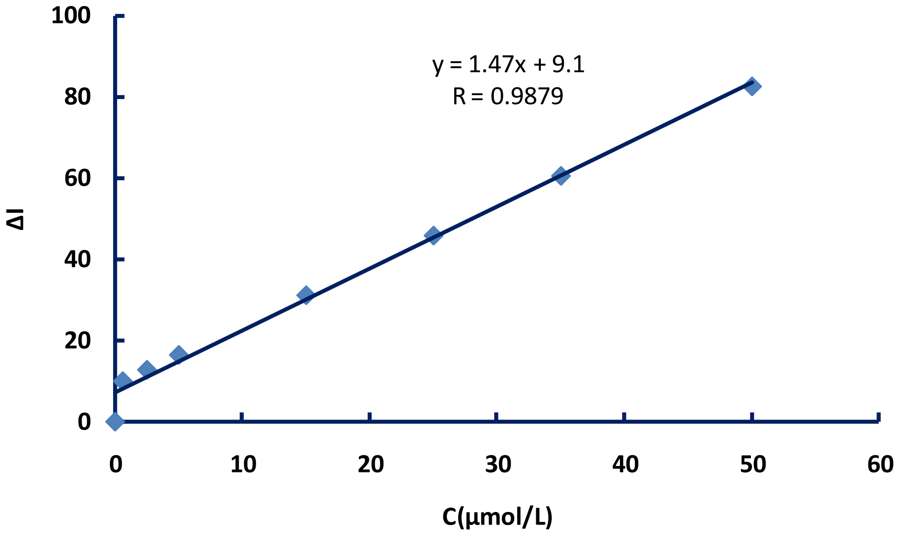

Figure S25 Working curve for SERS detection of AuNP$_b$ with safranine T probe

4.48μmol/L $HAuCl_4$-0.67mmol/L HCl-3.33mmol/L H_2O_2-AuNP$_B$-6.7mmol/L safranine T.

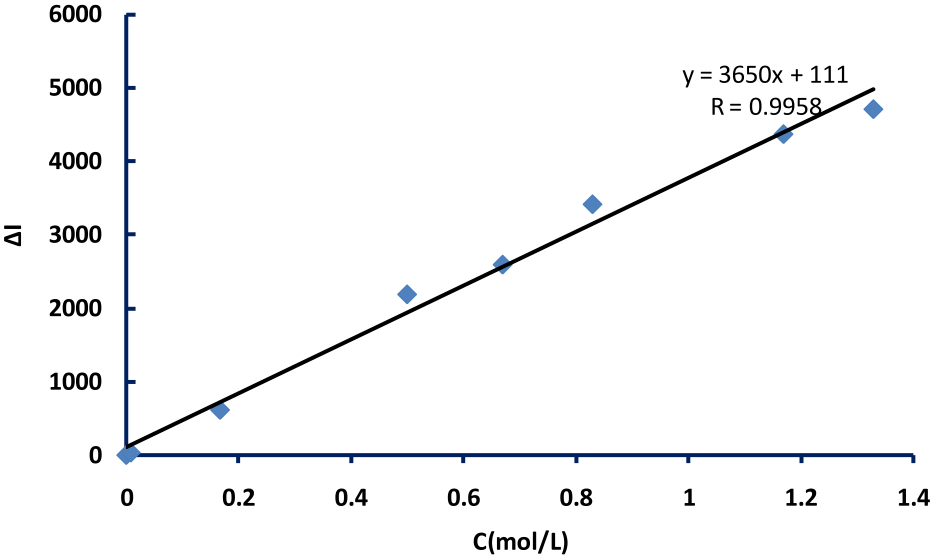

Figure S26 Working curve for RRS detection of Hg^{2+}.

4.48μmol/L $HAuCl_4$+0.67mmol/L HCl+3.33mmol/L H_2O_2-38ng/mL AuNP$_B$-Hg^{2+}

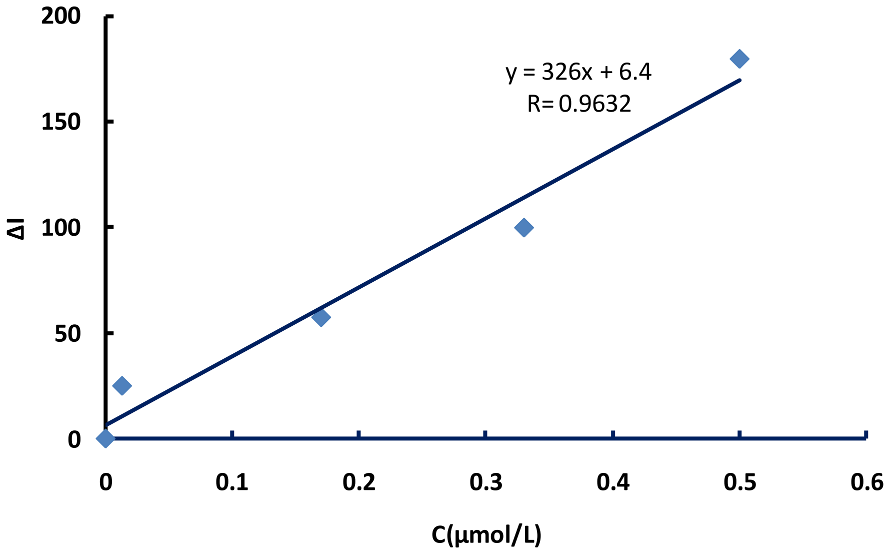

Figure S27 Working curve for SERS detection of Hg^{2+}.
4.48μmol/L $HAuCl_4$+0.67mmol/L HCl+3.33mmol/L H_2O_2-38ng/mL $AuNP_B$-1.3μmol/L VBB -Hg^{2+}

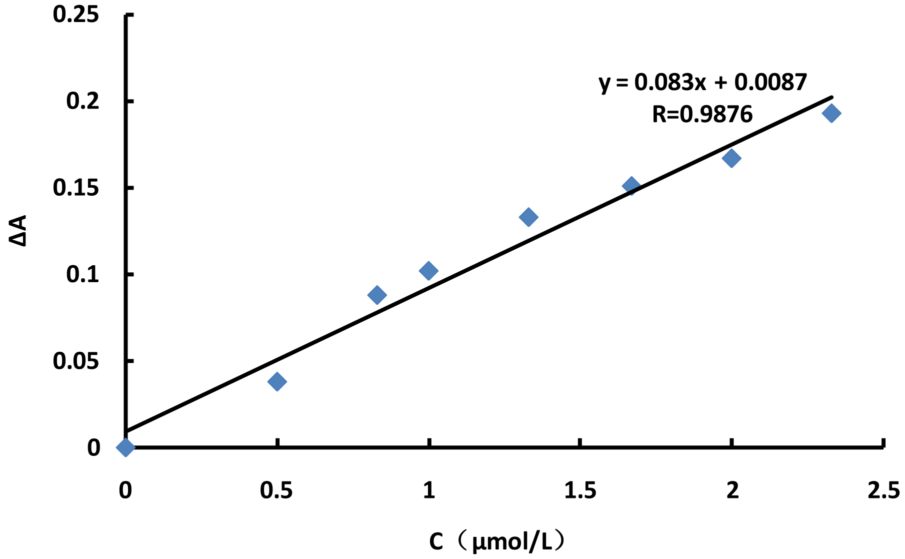

Figure S28 Working curve for Abs detection of Hg^{2+}.
4.48μmol/L $HAuCl_4$+0.67mmol/L HCl+3.33mmol/L H_2O_2-38ng/mL $AuNP_B$-Hg^{2+}

Table S1 Results for the determination of Hg^{2+} in water samples (n=5)

Sample	Hg^{2+} content （nmol/L）	Added Hg^{2+} （nmol/L）	Found Hg^{2+} (nmol/L)	Recoery （%）	RSD （%）	AAS （nmol/L）
Tap	13.8	10	10.2	102	5.2	13.4
River	16.8	10	9.70	97.0	4.8	17.2
Pond	21.5	10	9.85	98.5	4.5	23.1
